# PPSampler2: Predicting protein complexes more accurately and efficiently by sampling

**DOI:** 10.1186/1752-0509-7-S6-S14

**Published:** 2013-12-13

**Authors:** Chasanah Kusumastuti Widita, Osamu Maruyama

**Affiliations:** 1 Graduate School of Mathematics, Kyushu University, Fukuoka, 819-0395, Japan; 2 Institute of Mathematics for Industry, Kyushu University, Fukuoka, 819-0395, Japan

## Abstract

The problem of predicting sets of components of heteromeric protein complexes is a challenging problem in Systems Biology. There have been many tools proposed to predict those complexes. Among them, PPSampler, a protein complex prediction algorithm based on the Metropolis-Hastings algorithm, is reported to outperform other tools. In this work, we improve PPSampler by refining scoring functions and a proposal distribution used inside the algorithm so that predicted clusters are more accurate as well as the resulting algorithm runs faster. The new version is called PPSampler2. In computational experiments, PPSampler2 is shown to outperform other tools including PPSampler. The F-measure score of PPSampler2 is 0.67, which is at least 26% higher than those of the other tools. In addition, about 82% of the predicted clusters that are unmatched with any known complexes are statistically significant on the biological process aspect of Gene Ontology. Furthermore, the running time is reduced to twenty minutes, which is 1*/*24 of that of PPSampler.

## Background

Protein complexes are essential molecular entities in the cell because intrinsic functions of an individual protein are often performed in the form of a protein complex. Thus, it is helpful to identify all protein complexes of an organism for elucidation of the molecular mechanisms underlying biological processes. However, reliable protein complex purification experiments are rather laborious and time-consuming. Thus it has been expected to provide reliable candidates for true protein complexes by computational prediction methods.

Most computational approaches to predict the components of protein complexes are designed based on the observation that densely connected subgraphs in a protein-protein interaction (PPI) network are often overlapped with some known protein complexes. One of the differences among those methods is the search strategies to find good clusters of proteins. For example, MCL [1] is considered to be a clustering algorithm, in which clusters are formed by repeatedly executing an inflation step and a random walk step. RRW [2] and NWE [3] execute random walks with restarts and generate predicted protein clusters using the resulting stationary probabilities of the random walks. Note that the stationary probability from a protein to another which are both within a densely connected subgraph is likely to be high. COACH [4] finds extremely dense subgraphs which are called cores, and predicts protein clusters by extending cores with additional proteins out of the cores.

Our previous method, PPSampler [5], is designed based on the Metropolis-Hastings algorithm, in which a partition of all proteins is generated as a sample according to the probability distribution which is specified by a scoring function of a partition of all proteins. The entire scoring function consists of the following three scoring functions denoted by *f*_1_, *f*_2_, and *f*_3_. The main part of *f*_1 _is equivalent to the total sum of the PPI weights within predicted clusters of size two or more. The second scoring function of *f*_2 _is designed based on the constraint that the frequency of sizes of predicted clusters obeys a power-law distribution. This constraint is derived from the observation that the frequency of sizes of known complexes obeys a power-law distribution in CYC2008 [6] and CORUM [7], which are databases of protein complexes of yeast and human, respectively. Thus *f*_2 _evaluates the difference between a given power-law distribution and the distribution of sizes of clusters in a partition. The third scoring function of *f*_3 _is the gap between the number of proteins within predicted clusters of size two or more and a target value of that number. It should be noted here that *f*_2 _and *f*_3 _can be considered to be regularization terms to encourage sparse structures (see, for example, [8] for sparse structure). PPSampler is reported to outperform other prediction methods, including MCL, RRW, NWE, and COACH, in our previous work [5]. Especially, the F-measure score of PPSampler is 0.536, which is at least 30% better than those of the other methods.

In this paper, at first, we have improved the scoring functions, *f*_1_*, f*_2_, and *f*_3_, of PPSampler in order to predict protein complexes more accurately. The first scoring function of *f*1 is refined by replacing the sum of the weights of PPIs within a cluster with a generalized density of the cluster. The remaining scoring functions, *f*_2 _and *f*_3_, are also newly modeled, using Gaussian distributions. The resulting scoring functions are called *g*_1_, *g*_2_, and *g*_3_, respectively. Notice that *g*_2 _and *g*_3 _are also regularization terms to encourage sparse structures. Secondly, the new entire scoring function is formulated as the negative of the sum of *g*_1_, *g*_2_, and *g*_3 _although that of PPSampler is the negative of the product of *f*_1_*, f*_2_, and *f*_3_. Lastly, the proposal distribution, which proposes a candidate state given a current state, is also improved to enable a more efficient random walk over the states. Note that the second and third modifications enable the algorithm to run faster.

The resulting method is called PPSampler2. Hereafter PPSampler is called PPSampler1 to distinguish clearly between it and PPSampler2. The F-measure score of PPSampler2 is 26% higher than that of PPSampler1. In addition, about 82% of the predicted clusters that are unmatched with any known complexes are statistically significant on the biological process aspect of Gene Ontology. Furthermore, the running time is drastically reduced from eight hours to twenty minutes. Interestingly, it turns out that the two new scoring functions of *g*_1 _and *g*_3 _make *g*_2_, the scoring function based on a power-law distribution, unnecessary in the sense that, without *g*_2_, PPSampler2 always returns almost the same results as with *g*_2_. This would be due to the effect of the generalized density of *g*_1_.

## Methods

### Search by sampling

**Figure 1:**
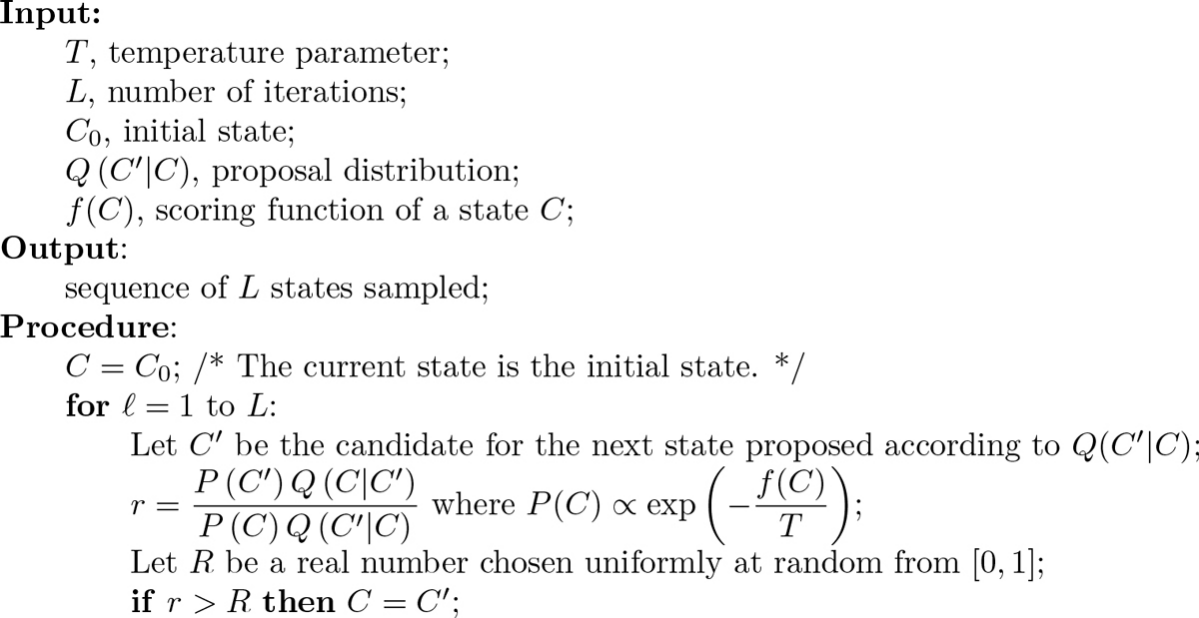
**Metropolis-Hastings algorithm**. Let *f*(*C*) be a scoring function of a state *C *whose optimal value is the minimum value of *f*. The Metropolis-Hastings algorithm generates *L *random samples from a probability distribution $P\left(C\right)\propto \text{exp}\left(-\frac{f\left(C\right)}{T}\right)$, where *T *is a parameter called temperature.

PPSampler2 is designed based on the Metropolis-Hastings algorithm [9] (see Figure 1), which is a Markov chain Monte Carlo (MCMC) method [10]. MCMC is known as a class of algorithms for generating samples from particular probability distributions. Suppose that *f*(*C*) is a scoring function to be minimized, where *C *is a state in a domain of states, *D*. A target probability distribution is determined from *f*(*C*) by the following way:

$$P\left(C\right)\propto \text{exp}\left(-\frac{f\left(C\right)}{T}\right),$$

where *T *is a temperature parameter. Note that there exists the relationship that the higher the probability, the lower the score. Thus, by sampling, a minimized score and the corresponding state can be found. To exploit the Metropolis-Hastings algorithm, in addition to *D *and *f*(*C*), a proposal distribution, denoted by *Q*(*C*′|*C*), which is a probability distribution of *C*′ ∈ *D *given *C *∈ *D*, should be also specified. The formulations of them for PPSampler2 are given in the subsequent sections after that of a PPI dataset.

### Weighted protein-protein interaction network

A PPI network is often used as an input to protein complex prediction tools. It can be defined as an undirected graph, *G *= (*V, E*), where *V *is a set of proteins under consideration, and *E *is a subset of *V *× *V *\ {{*u, u*}|*u *∈ *V*}, representing a set of PPIs. Notice that any self-interactions, {*u, u*}, are excluded in *E*. Suppose that each PPI, *e*, has a weight, *w*(*e*) ∈ ℝ_+_, representing the reliability of the interaction of *e*. Note that the higher the weight of an interaction, the more reliable the interaction. For a pair of proteins, *e*, not in *E*, the weight of *e *is defined as *w*(*e*) = 0.

### States

The domain of states we use here is the same as in [5], which can be explained as follows. Let *C *be a partition of *V *. Namely, *C *can be represented as

$$C\;=\;\left\{c_{1},\;\ldots \;,\;c_{n}\subseteq V\;\;\left|\begin{array}{c} \forall i,\;c_{i}\;\neq \;\emptyset \\ \cup_{1\leq i\leq n}c_{i}\;=\;V,\\ \forall i,j\left(\neq i\right),\;c_{i}\cap c_{j}=\emptyset \end{array}\right)\right\}.$$

An element of *C *is called a cluster of proteins. All partitions of *V *are states in *D*. In the subsequent section, the formulation of the score of *C *is given explicitly.

### Scoring functions

In our previous work [5], the entire scoring function, which is denoted by *f *′(*C*) in this paper, for a partition *C *is formulated as the negative of the product of three different scoring functions, *f*_1_(*C*), *f*_2_(*C*), and *f*_3_(*C*), *i.e., f *′(*C*) = −*f*_1_(*C*)·*f*_2_(*C*)·*f*_3_(*C*). These three scoring functions are formulated as follows. The first scoring function of *f*_1 _is designed to return the total sum of the PPI weights within predicted clusters of size two or more in *C*. The second scoring function of *f*_2 _evaluates the difference between a given power-law distribution and the distribution of sizes of clusters in *C*. The third scoring function of *f*_3 _is the gap between the number of proteins within predicted clusters of size two or more in *C *and a target value of that number.

The new entire scoring function, *f*(*C*), proposed in this work is given as the negative of the *sum *of three new scoring functions, *g*_1_(*C*), *g*_2_(*C*), and *g*_3_(*C*), *i.e.*,

$$f\left(C\right)=-\left(g_{\text{1}}\left(C\right)\;+\;g_{\text{2}}\left(C\right)\;+\;g_{\text{3}}\left(C\right)\right).$$

As can be seen, *f *is changed from the product of three terms in the previous work. The motivation is to increase the acceptance rate of proposed states. For current and candidate states, *C *and *C*′, the term of −(*f*(*C*′) − *f*(*C*)), which is calculated in the Metropolis-Hastings algorithm, can be expected to be higher than −(*f *′(*C*′) − *f *′(*C*)) due to the difference between the forms of *f *and *f *′.

The three new scoring functions, *g*_1_(*C*), *g*_2_(*C*) and *g*_3_(*C*), use the same source data as *f*_1_(*C*), *f*_2_(*C*) and *f*_3_(*C*), respectively, but are refined in the following way.

*Scoring function g*_1_(*C*)

We define $g_{\text{1}}\left(C\right)\;=\underset{c\in C}{\sum }g_{1}\left(c\right)$ where

$$g_{1}\left(c\right)=\left\{\begin{array}{cc} 0 & \text{if |}c\text{| = 1},\\ -\infty & \text{else if |}c\text{|}>N\text{ or}\\ & \;\;\;\exists u\in c,\forall \upsilon \left(\neq u\right)\in c,\\ & \;\;\;w\left(\left\{u,\upsilon \right\}\right)=0,\\ \underset{u,\upsilon \left(\neq u\right)\in c}{\sum }\frac{w\left(u,\upsilon \right)}{\sqrt{\left|c\right|}} & \text{otherwise,} \end{array}\right)$$

where *N *is a parameter specifying the upper bound on the size of a cluster in *C*. The above function, *g*_1_(*c*), can be interpreted as follows. If *c *is of size one, *g*_1_(*c*) is set to be zero. This means that *c *has no influence to *g*_1_(*C*). Next, *g*_1_(*c*) is negative infinity if the size of *c *is greater than *N*, or if *c *includes a protein which has no interactions with the other proteins in *c*. In this case, *P*(*C*) goes to zero. Otherwise, *g*_1_(*c*) is equal to the total sum of the weights of all interactions within *c *divided by the positive square root of the size of *c*.

Note that the scoring function of the previous work [5] corresponding to *g*_1_(*c*) is *f*_1_(*c*). The difference between *g*_1_(*c*) and *f*_1_(*c*) appears only in the last case of the three cases, in which the score of a cluster, *c*, is formulated as $\underset{u,v\left(\neq u\right)\in c}{\sum }w\left(u,\;v\right)$ in the previous work. If it is furthermore divided by the factor of $\sqrt{\left|c\right|}$, the resulting term is equivalent to the scoring function defined above, $g_{1}\left(c\right)\;=\;\underset{u,v\left(\neq u\right)\in c}{\sum }\frac{w\left(u,\;v\right)}{\sqrt{\left|c\right|}}.$

The new scoring function, *g*_1_(*c*), can be considered to be a *density *measure. Actually, density measures are used in many previous works to infer protein complexes. For example, Wu *et al*. [4] uses a typical density measures $x/\frac{|c|\left(|c|-1\right)}{2}$, where *x *is the number of interactions within *c*. Namely, its denominator, $\frac{|c|\left(|c|-1\right)}{2}$, is equivalent to the possible maximum number of edges in a subgraph with |*c*| nodes. Note that because the PPI network used in their work is supposed to be unweighted, the numerator is just the number of edges in

*c*. However, it can be observed that the larger a cluster, the relatively lower the value of the above measure. Namely, the larger a cluster, the severer evaluation it suffers. Then, Feng *et al*. [11] eased this peculiarity by adopting |*c*| as the denominator instead of $\frac{|c|\left(|c|-1\right)}{2}$. Namely, the resulting measure is $\frac{x}{\left|c\right|}$. In this work, in addition to the denominators mentioned above, which are $\frac{|c|\left(|c|-1\right)}{2}$ and |*c*|, more gradual functions, $\sqrt{\left|c\right|}$ and log_2_ |*c*|, have also been evaluated. Then, it turns out that $\sqrt{\left|c\right|}$ and log_2_ |*c*| give similar F-measure scores which are higher than those of the others. Thus, $\sqrt{\left|c\right|}$ is selected as the denominator in PPSampler2.

*Scoring function g*_2_(*C*)

The second scoring function, *g*_2_(*C*), evaluates how much closer the relative frequency of clusters of a size in *C *and a predefined target relative frequency of the size, which is given as a parameter. We denote by *ψ_C_*(*i*) the relative frequency of clusters of size *i *in *C *for size *i *= (2, 3*, . . . , N *). In addition, we denote by *ψ*(*i*) a predefined target value of the relative frequency of clusters of size *i *in *C*. Note that *ψ*(*i*) is set to be a power-law function

$$\frac{1}{\sum_{j=2}^{N}j^{-\gamma }}.\;i^{-\gamma }$$

where *γ *is the power-law parameter and its default value is set to be 2. This default value is an approximation of the value, 2.02, of the regression curve obtained from the relative frequency of CYC2008 complexes of size *i *= (2, 3*, . . . , N *) by minimizing the sum of squared errors at sizes *i*. The sum of the squared errors is small (0.0014).

For a cluster size, *i, ψ_C_*(*i*) is evaluated by the normal distribution of mean *ψ*(*i*) and standard deviation *σ*_2*,i*_, which defined by the formula:

$$p\left(\psi_{C}\left(i\right)|\psi \left(i\right),\;\sigma_{\text{2},i}\right)\;\;\;\propto \;\;\text{exp}\left\{-\frac{{\left(\psi_{C}\left(i\right)\;-\psi \left(i\right)\right)}^{2}}{2\sigma_{2,i}^{2}}\right\}.$$

The joint probability of *ψ_C_*(2)*, ψ_C_*(3)*, . . . , ψ_C_*(*N *) given *ψ*(2)*, ψ*(3)*, . . . , ψ*(*N *) and ***σ***_2 _= (*σ*_2,2_, *σ*_2,3_, . . . , *σ*_2*,N *_) is formulated as the product of the above *N *− 1 formulas:

$$\begin{array}{cc} p\left(\psi_{C}\left(2\right),\psi_{C}\left(3\right),\ldots ,\psi_{C}\left(N\right)|\psi ,\sigma_{2}\right) & \propto \overset{N}{\underset{i=2}{\prod }}\text{exp}\left\{-\frac{{\left(\psi_{C}\left(i\right)-\psi \left(i\right)\right)}^{2}}{2\sigma_{2,i}^{2}}\right\}\\ & =\text{ exp}\left\{-\overset{N}{\underset{i=2}{\sum }}\frac{{\left(\psi_{C}\left(i\right)-\psi \left(i\right)\right)}^{2}}{2\sigma_{2,i}^{2}}\right\}\\ & =\text{ exp}\left\{g_{2}\left(C\right)\right\} \end{array}$$

where

$${\text{g}}_{2}\left(C\right)\;\text{ = }-\overset{N}{\underset{i=2}{\sum }}\frac{{\left(\psi_{C}\left(i\right)-\psi \left(i\right)\right)}^{2}}{2\sigma_{2,i}^{2}}.$$

**Figure 2:**
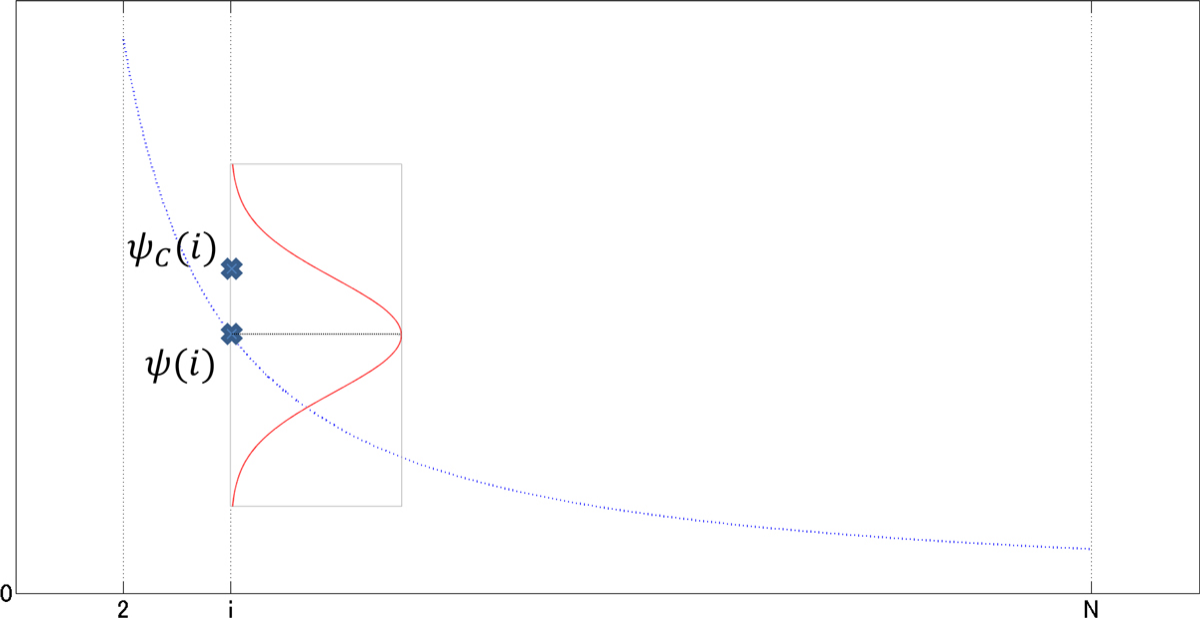
**Scoring function, *g*_2_(*C*)**. For each cluster size, *i *= 2, 3*,..., N , ψ_C_*(*i*) is evaluated by the Gaussian distribution of mean *ψ*(*i*) and standard deviation *σ*_2*,i*_, which is shown sideways on the vertical line of size *i*.

In this way, the function of *g*_2_(*C*) is derived (see Figure 2). Note that the term corresponding to the normalizing constant is omitted because it is a constant for any *C*. In this work, $\sigma_{2,i}^{2}$ is set to be 1000 × 1.1^−*i*^. This implies that the larger a cluster, the more severely it is evaluated in *g*_2_.

*Scoring function g*_3_(*C*)

The third scoring function, *g*_3_(*C*), is also derived from a normal distribution. The number of proteins within the clusters of size two or more in *C *is denoted by *s*(*C*), i.e.,

$$s\left(C\right)=\underset{c\in C\;\text{s}.\text{t}.\;\left|c\right|\geq \text{2}}{\sum }\left|c\right|.$$

Let *λ *be a target value of *s*(*C*). The value of *s*(*C*) is evaluated by the normal distribution of mean *λ *and standard deviation *σ*_2_,

$$\begin{array}{cc} p\left(s\left(C\right)|\lambda ,\;\sigma_{\text{2}}\right) & \propto \text{exp}\left\{-\frac{{\left(s\left(C\right)-\lambda \right)}^{\text{2}}}{\text{2}\sigma_{\text{2}}^{2}}\right\}\\ & =\;\text{exp}\left\{g_{\text{3}}\left(C\right)\right\} \end{array}$$

where

$$g_{\text{3}}\left(C\right)=-{\frac{\left(s\left(C\right)\;-\;\lambda \right)}{\text{2}\sigma_{\text{2}}^{2}}}^{2}.$$

In this work, $\sigma_{2}^{2}$ is set to be 10^6^.

### Proposal distribution

A proposal distribution, *Q*(*C*′|*C*), provides the transition probability to a candidate state *C*′∈ *D *given a current state *C *∈ *D*. The proposal distribution we use here is obtained by improving that of PPSampler1. The differences between them will be pointed out in the following explanation of our new proposal distribution.

At first, a protein, *u *∈ *V*, is chosen uniformly at random. Thus, the probability of choosing *u *is $\frac{1}{\left|V\right|}$.The randomly chosen protein *u *is removed from the cluster including *u *in *C*, and then the destination of *u *is determined by the following probabilistic procedure. As a result, a conditional probability is associated with the resulting state.

(i) With a constant probability *β*, the chosen protein, *u*, forms a new singleton cluster. The resulting state *C*′ has the following conditional probability

$$Q\left(C^{\prime }|C\right)=\frac{\beta }{\left|V\right|}.$$

(ii) Suppose that the chosen protein, *u*, is added to an existing cluster in *C*, which is chosen probabilistically by the following way. All the proteins *v *∈ *V *\ {*u*} that share interactions with *u *are sorted according to the weights *w*({*u, v*}) in decreasing order. The *i*th protein in the resulting list is denoted by *v_i_*. Namely, we have the following relationship:

$$w\left(\left\{u,\;v_{\text{1}}\right\}\right)\;\geq \;w\left(\left\{u,\;v_{\text{2}}\right\}\right)\;\geq \;\ldots \;\geq w\left(\left\{u,\;v_{d_{u}}\right\}\right),$$

where *d_u _*is the degree of *u *in the PPI network. Note that in the proposal distribution of PPSampler1, all proteins *v *∈ *V *\ {*u*} are sorted and used in the subsequent procedure. In the next step, the probability of choosing a cluster, *c *∈ *C*, is set to be the probability proportional to

$$\underset{i\;\text{s}.\text{t}.\;v_{i}\in c,\left\{u,v_{i}\right\}\in E}{\sum }\frac{1}{i^{\text{2}}}.$$

Note that the term corresponding to $\frac{1}{i^{2}}$ is $\frac{1}{i}$ in PPSampler1. As a result, the resulting proposal distribution in this case is

$$Q\left(C^{\prime },|,C\right)\propto \frac{\text{1}-\beta }{\left|V\right|}\underset{i\;\text{s}.\text{t}.\;v_{i}\in c,\left\{u,v_{i}\right\}\in E}{\sum }\frac{1}{i^{\text{2}}}.$$

The value of *β *is set to be *β *= 1*/*100 as in [5].

Notice that the reduction of the running time of PPSampler2 is realized by the combination of the two factors. A factor is that the scoring function, *f*, is changed from the product of three terms to the sum of them. Another is that the new proposal distribution proposes states which are likely to have higher probabilities.

### Initial state

The initial state is the same as that of PPSampler1, which is the following partition. Let *u *and *v *be the pair of proteins with the highest PPI weight among all of the given PPI weights. Then the cluster consisting only of *u *and *v *is created. In addition to it, each of the remaining proteins forms a singleton cluster. It is trivial that the probability of this state is not zero.

### Output of PPSampler2

PPSampler2 returns as output the state, *C*, with the highest probability among all the states sampled. After removing all the clusters of size one in *C*, the remaining clusters are all treated as predicted complexes.

### Matching statistics

#### Performance measures

To evaluate predicted clusters by known complexes, three measures, precision, recall, and F-measure are used. To use the three measures, a matching criterion for two sets of proteins is required to determine whether a predicted cluster is matched with a known complex. For two sets of proteins, *s *and *t*, the *overlap ratio *between *s *and *t*, denoted by *ov*(*s, t*), is formulated as follows:

$$ov\left(s,t\right)=\left\{\begin{array}{c} \frac{\left|s\cap t\right|}{\sqrt{\left|s\right|\cdot \left|t\right|}}\\ 0 \end{array}\right)\;\;\;\;\;\;\;\;\;\;\;\begin{array}{c} \text{if }\left|s\cap t\right|\geq 2\\ \text{otherwise}. \end{array}$$

Thus, it is one if *s *and *t *are identical to each other. We say that *s *and *t *are matched if *ov*(*s, t*) ≥ *η*, where *η *is a predefined threshold. Notice that, in the case where *s *and *t *share only one protein, the overlap ratio turns to be zero. Otherwise, the overlap ratio is equal to the ratio of the number of common proteins between *s *and *t *to the geometric mean of the sizes of *s *and *t*.

On the other hand, if *s *and *t *share less than two proteins, the overlap ratio is defined as zero. The reason to do that can be explained as follows. The typical value of *η *in the literature is $\sqrt{0.\text{2}}=0.\text{4472}$ (see, for example, [12]). However, with this threshold, if *s *and *t *are both of size two and share only one protein, they are determined to be matched because $\frac{\text{1}}{\sqrt{\text{2}\cdot \text{2}}}=0.\text{5}>0.\text{4472}.$ Notice that this case tends to happen by chance. Suppose that there are many known complexes of size two. In this situation, by predicting many clusters of size two, a known complex of size two can be matched with such a predicted cluster by sharing only one protein. The overlap ratio define here is designed to avoid this unfavorable situation. Note that *η *is set to be $\sqrt{0.2}$ in this work.

Here we suppose that *C *is a set of predicted clusters of proteins by a protein complex prediction algorithm and *K *is a set of known complexes. The *precision *of *C *to *K *with *η *is defined as

$$precision\left(C,K,\eta \right)=\;\frac{N_{pc}\left(C,K,\eta \right)}{\left|C\right|},$$

where

$$N_{pc}\left(C,\;K,\;\eta \right)\;=\;\left|\{c\right|c\in C,\exists k\in K,\;ov\left(c,\;k\right)\;\geq \;\eta \}|,$$

which is the number of predicted clusters in *C *matched with at least one known complex in *K *with an overlap ratio threshold *η*. In a similar way, the *recall *of *C *to *K *with *η *is defined as

$$recall\left(C,K,\eta \right)\;=\;\frac{N_{kc}\left(C,K,\eta \right)}{\left|K\right|},$$

where

$$N_{kc}\left(C,\;K,\;\eta \right)=\left|\{,k\right|k\in K,\exists c\in C,\;ov\left(k,\;c\right)\;\geq \;\eta \}|,$$

which is the number of known complexes in *K *matched with at least one predicted cluster in *C *with an overlap ratio threshold *η*. The *F-measure *of *C *to *K *with *η *is defined as the harmonic mean of the corresponding precision and recall. Namely, we have

$$F\left(C,K,\eta \right)=\text{2}\text{.}\frac{precision\left(C,K,\eta \right)\cdot recall\left(C,K,\eta \right)}{precision\left(C,\;K,\;\eta \right)\;+\;recall\left(C,K,\eta \right)}.$$

Notice that all clusters of size one are completely not counted in this matching statistics. Hereafter, a predicted cluster of any tool means a set of two or more proteins predicted as a protein complex.

#### Statistical significance by Gene Ontology

The Gene Ontology (GO) provides a unified representation of gene and gene product attributes across all species [13]. Thus, GO is often exploited to find some biological coherence of a newly found group of proteins, like functional modules and protein complexes. For a predicted cluster, if a more specific GO term annotates more proteins in the predicted cluster, the term would be a better biological characterization of the cluster.

Such a term can be identified by comparing the statistical significances of a cluster by GO terms. The statistical significance of a predicted cluster, *c*, by a GO term, *t*, can be calculated by the hypergeometric distribution in the following equation:

$$p-value\;=\;1-\overset{b-\text{1}}{\underset{i=0}{\sum }}\frac{\left(\begin{array}{c} \left|M\right|\\ i \end{array}\right)\left(\begin{array}{c} \left|V|-|M\right|\\ |c|-i \end{array}\right)}{\left(\begin{array}{c} \left|V\right|\\ \left|c\right| \end{array}\right)}$$

where *c *contains *b *proteins in the set, *M*, of proteins annotated by *t*, and *V *is the set of all proteins in the whole PPI network [14]. In this work, the p-values (with Bonferroni correction) of predicted clusters are calculated by the tool, "Generic gene ontology (GO) term finder" (http://go.princeton.edu/cgi-bin/GOTermFinder), whose implementation depends on GO::TermFinder [15]. The p-value cutoff used in this work is set to be the default value of 0.01.

## Result

In this section, we report the results of performance comparison, carried out in a similar way as [5], of PPSampler2 with the following public tools, MCL [16], MCODE [12], DPClus [17], CMC [18], COACH [4], RRW [2], NWE [3], and PPSampler1 [5]. The outputs of these algorithms are evaluated by the known protein complexes of CYC2008 and GO terms.

### Materials

The set of all PPIs with their weights in WI-PHI [19] is given as input to the above algorithms. It contains 49607 non-self-interactions with 5953 proteins (393 self interactions are excluded). The average degree of the proteins is 16.7. Every interaction of them is assigned a weight representing the reliability of the interaction. The weight of an interaction is determined from datasets derived from high-throughput assays, including tandem affinity purification coupled to mass spectrometry (TAP-MS) and the yeast two-hybrid system, and a literature-curated physical interaction dataset, which is used as a benchmark set. The log-likelihood of each dataset is calculated with the benchmark set. The weight of an interaction is formulated as the sum of, over those datasets, the product of the socio-affinity index [20] of the interaction on a dataset and the log-likelihood of the dataset. The resulting weights are ranged from 6.6 to 146.6. The higher the weight of an interaction, the more reliable. Note that among the above algorithms, MCL, RRW, NWE, PPSampler1, and PPSampler2 exploit the weights, and the others do not.

The gold standard dataset of known complexes used here is the complexes of the CYC2008 database [6]. Recall that this database have 408 curated heteromeric protein complexes of *S. cerevisiae*. It is pointed out in our previous work [5] that among those complexes, 172 (42%) and 87 (21%) are hetero-dimeric and trimeric complexes, respectively.

### Configuration setting

The default values of the parameters of PPSampler2 is given in Table 1. Later, the robustness of PPSampler2 to some of those parameters will be examined. With the default parameter set, ten executions of PPSampler2 have been carried out. To compare performance, the three performance measures, precision, recall, and F-measure, are calculated. For PPSampler2, they are averaged over ten executions. All the results of the other methods, except those for predicted clusters of size four or more, are obtained from [5]. Recall that the threshold, *η*, of the overlap ratio is set to be $\sqrt{0.\text{2}}=0.\text{4472}$. Note that PPSampler2 normalizes PPI weights by dividing by the maximum one.

**Table 1:** Default parameter values of PPSampler2.

Parameter	notation & value
Temperature	*T *= 10^−9^
number of iterations	*L *= 2 × 10^6^
maximum cluster size	*N *= 100
probability of making a new single cluster	*β *= 0.01
parameters of *g*_2_	*γ *= 2
	$\sigma_{\text{2},i}^{2}=\text{1}000\;\times \;\text{1}.{\text{1}}^{-i}$
parameters of *g*_3_	*λ *= 2000
	$\sigma_{3}^{\text{2}}\;=\;10^{6}$

### Performance comparison for all predicted clusters

**Figure 3:**
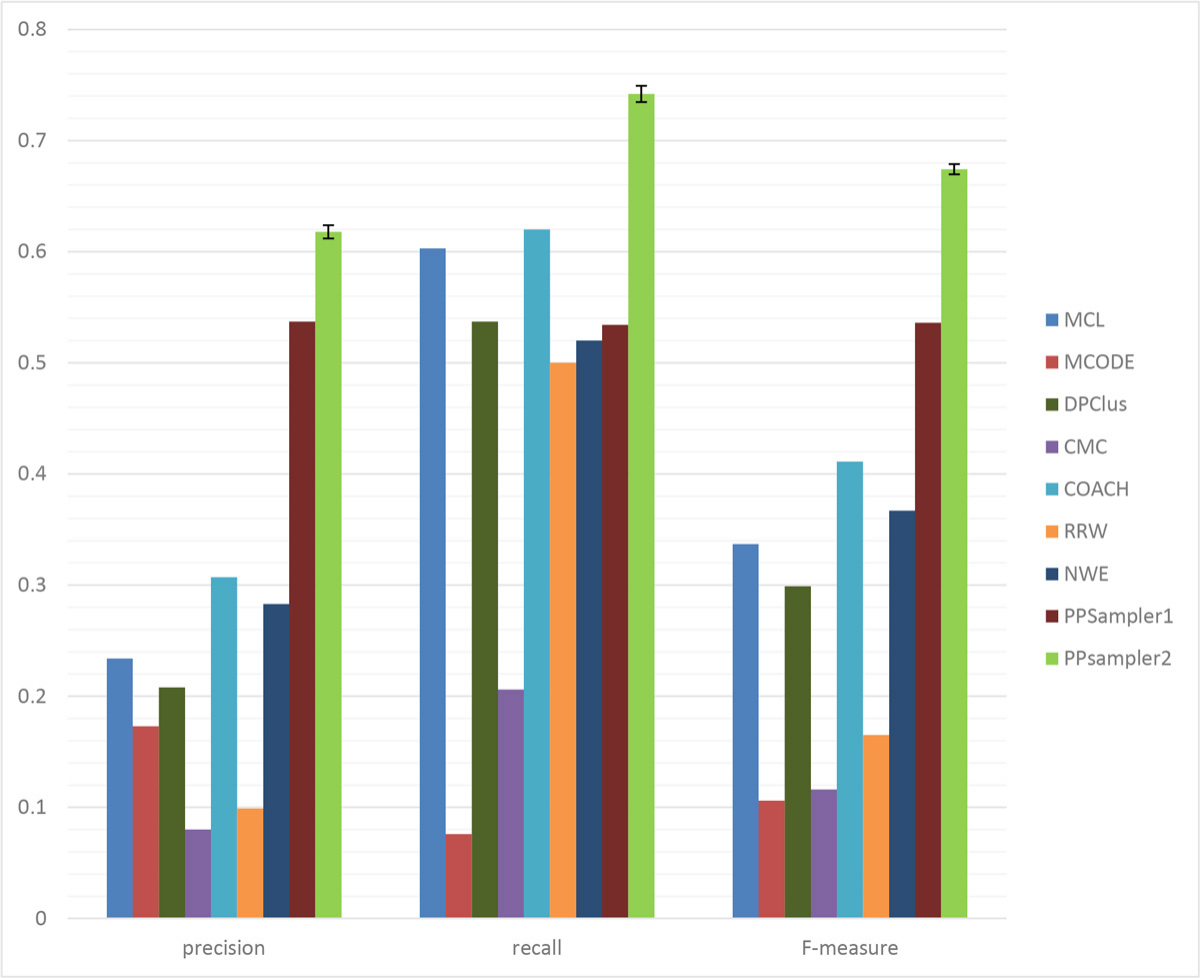
**Performance comparison for all predicted clusters**. The error bars of PPSampler2 show the standard deviations.

At first, all the predicted clusters of size two or more are evaluated with all the known protein complexes. The matching results of the above algorithms are shown in Table 2. The row of #protein gives the number of proteins within predicted clusters. It can be seen that the number varies with the individual algorithm. It ranges from 1626 to 5869, which are 27.3% and 98.6% of the total number of proteins of WI-PHI (5953), respectively. By this measure, the algorithms can be grouped into two groups. One is the group using many proteins over 4000, which includes MCL, DPClus, CMC, COACH, and RRW. The other group, including MCODE, NWE, PPSampler1, and PPSampler2, uses about 2000 proteins. The row of #cluster shows the number of clusters predicted by each algorithm. It also varies with the individual algorithm. The lowest value is 156 given by MCODE, and followed by 350 (PPSampler1), 402 (PPSampler2), and 720 (NWE). The other tools have about one thousand or more clusters. The row of Avg. size provides the mean of the sizes of predicted clusters. The algorithms can be classified into three groups. One is the group of RRW and NWE whose average size is about two. The second one is the middle-sized group, including MCL, DPClus, PPSampler1, and PPSampler2. The size is about five to six. The large-sized group consists of MCODE, CMC, and COACH. The last two rows show the values of *N_pc _*and *N_kc_*, respectively. Those numbers lead to precision, recall, and F-measure, which are graphically represented in Figure 3.

**Table 2:** The matching results of all predicted clusters.

	MCL	MCODE	DPClus	CMC	COACH	RRW	NWE	PPSampler1	PPSampler2
#protein	5869	2432	4888	5868	4094	4240	1626	2001	2009.90 ± 0.30
#cluster	880	156	925	978	1353	1984	720	350	402.10 ± 5.20
Avg. size	6.67	15.59	6.91	20.65	13.29	2.14	2.26	5.72	5.00 ± 0.06
*N_pc_*	206	27	192	79	416	196	204	188	248.40 ± 2.37
*N_kc_*	246	31	219	84	253	204	212	218	302.70 ± 3.00

#protein gives the number of proteins within predicted clusters. #cluster shows the number of predicted clusters. Avg. size provides the mean of the sizes of predicted clusters.

The (average) precision score of PPSampler2 is 0.618, which is 15% higher than the second best, given by PPSampler1 (0.537). In addition, the third best is 0.307, achieved by COACH, which is only 50% of the best. Thus, PPSampler2 outperforms the other algorithms in precision.

In recall, PPSampler2 outperforms the others, too. The recall score is 0.742, followed by 0.620 and 0.603 given by COACH and MCL, respectively. Thus, the best score is 20% and 23% higher than them, respectively. Note that the recall score of PPSampler2 is 39% higher than that of PPSampler1, 0.534.

In F-measure, PPSampler2 achieves the highest score, 0.674. It is 26% higher than the second highest, 0.536, given by PPSampler1. Note that PPSampler1 needs about eight hours to achieve that F-measure score. On the other hand, PPSampler2 can obtain its F-measure score in twenty minutes. Namely, PPSampler2 runs 24 times faster than PPSampler1. Thus, PPSampler2 is superior to PPSampler1 in prediction accuracy as well as running-time. Furthermore, the third highest F-measure score, achieved by COACH, is 0.411. Thus the F-measure of PPSampler2 is 64% higher than it. This indicates how high PPSampler2 outperforms the others.

It would be interesting to see which known complexes are successfully detected by PPSampler2. All of the known protein complexes perfectly detected by PPSampler2 and not by the other tools are extracted. For each of those known protein complexes, the best overlap ratio obtained by each algorithm is given in Additional file 1. The number of such complexes is 35, and the sizes of them are widely ranged from 2 to 25. Interestingly, MCL finds all of the complexes approximately but except the first one. This can be related to the common feature between MCL and PPSampler2 that the structure of their solutions is modeled as a partition of all proteins.

### Size-dependent performance comparisons

As mentioned before, it can be found that 172 (42%) of the 408 curated heteromeric protein complexes in the CYC2008 database are heterodimeric protein complexes, and 87 (21%) of them are heterotrimeric protein complexes. Totally, 259 (63%) of the 408 complexes are complexes of size two or three. They can be said to be the majority of the known protein complexes. Thus, the performances on those hetero-dimeric and trimeric complexes will be dominant in the performance on the set of the 408 complexes. Then the performances on those small-sized complexes are evaluated. In addition, the performance on the remaining predicted clusters, *i.e.*, those of size four or more is also considered, because many prediction algorithms have been evaluated by known complexes of size four (or three possibly) or more (see, for example, [3,21]). Thus, it is interesting to see how good the performance of PPSampler2 w.r.t. the range of sizes is.

The performance measures specialized for this purpose are almost the same as ones formulated in [5]. For the set, *C*, of all clusters predicted by an algorithm, we denote by *C*|*_i _*the subset of *C *whose elements are of size *i *and by *C*|_≥*i *_the subset of *C *whose elements are of size *i *or more. For the set, *K*, of all known protein complexes, we denote by *K*|*_i _*the subset of *K *whose elements are of size *i *and by *K*|_≥*i *_the subset of *K *whose elements are of size *i *or more. For each of the sizes of *i *= 2 and 3, the precision and recall for size *i *are defined as *precision $\left(C{|}_{i},\;K,\sqrt{0.\text{2}}\right)$*and *recall *$\left(C,\;K{|}_{i},\sqrt{0.\text{2}}\right)$, respectively. The corresponding F-measure is the harmonic mean of these precision and recall. In the similar way, the precision and recall for size four or more are defined as *precision $\left(C{|}_{\geq \text{4}},\;K,\sqrt{0.2}\right)$*and *recall $\left(C,\;K{|}_{\geq \text{4}},\sqrt{0.\text{2}}\right),$*respectively. Note that *K *is again set to be the set of all protein complexes in CYC2008.

**Figure 4:**
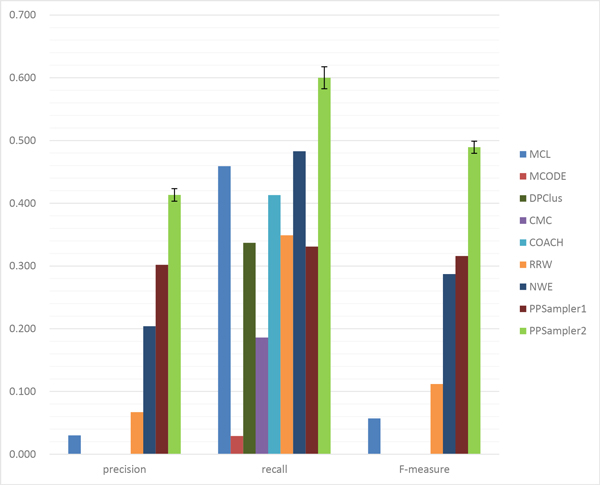
**Performance comparison on heterodimeric protein complexes**.

The matching results on size two is shown in Table 3. It can be seen that only several clusters of size two are predicted by MCODE, DPClus, and CMC, and that no clusters of size two are generated by COACH. This outcome is thought to be due to their strategies that put priority on generating reliable large clusters. The performance for this case is given in Figure 4. Clearly, PPSampler2 outperforms the others in precision, recall, and F-measure. Especially, in recall, PPSampler2 is significantly improved from PPSampler1, whose rank is seventh.

**Table 3:** The matching results on size two.

	MCL	MCODE	DPClus	CMC	COACH	RRW	NWE	PPSampler1	PPSampler2
#protein	462	6	2	12	0	3648	1264	258	219.20 ± 6.58
#cluster	231	3	1	6	0	1824	632	129	109.60 ± 3.29
*N_pc_*	7	0	0	0	0	122	129	39	45.30 ± 1.90
*N_kc_*	79	5	58	32	71	60	83	57	103.20 ± 3.03

**Figure 5:**
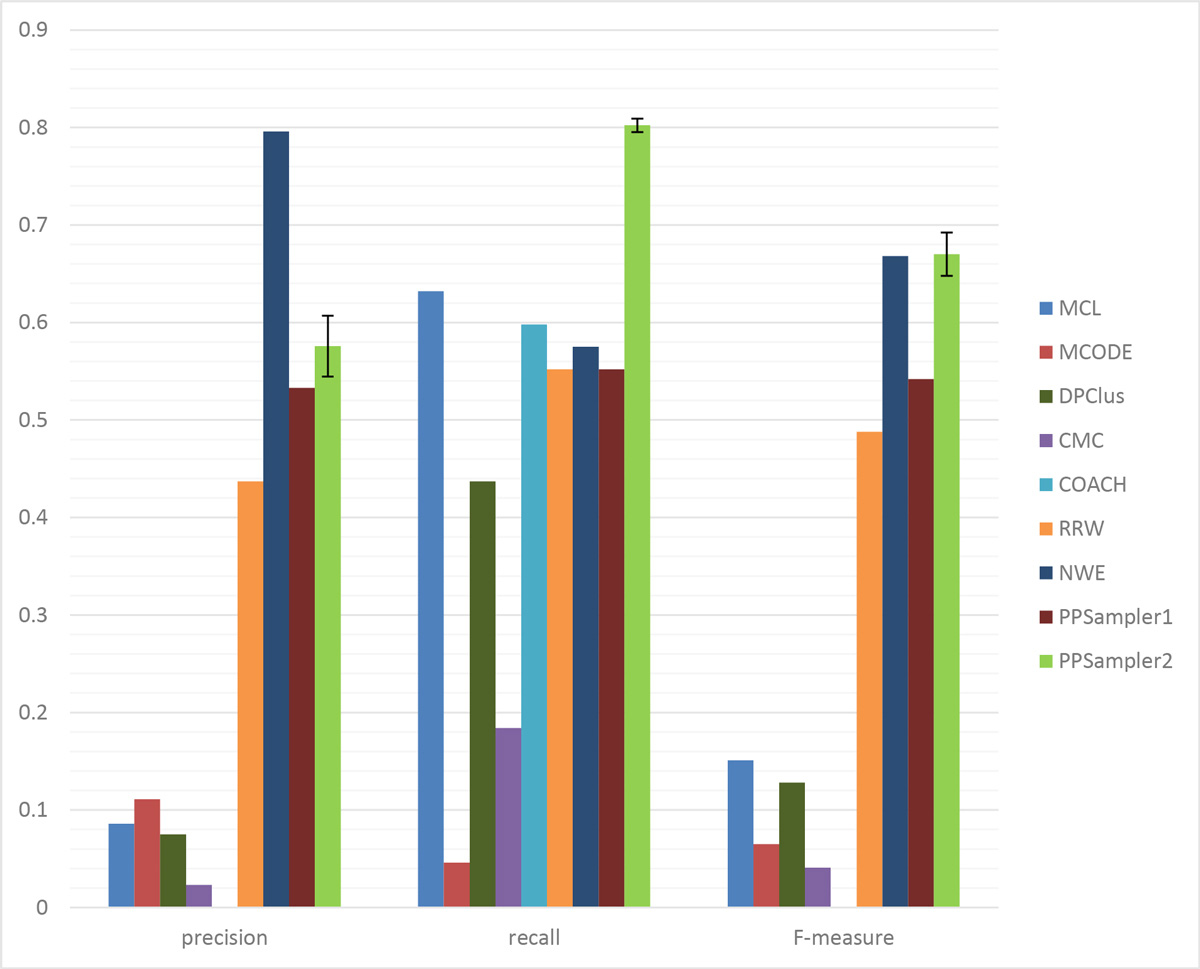
**Performance comparison on heterotrimeric protein complexes**.

The matching results on size three is shown in Table 4. More or less, all the algorithms predict clusters of size three. However, the four algorithms, MCODE, DPClus, CMC, and COACH, have low scores in *N_pc_*. This result is reflected directly to precision, shown in Figure 5. Their scores of precision are quite low. Notice that the four algorithms are known to predict less or no clusters of size two. Thus, they seem to be vulnerable to small-sized clusters. The best score of precision is achieved by NWE, followed by PPSampler2. In recall, PPSampler2 is the best performer, and the score of NWE is not high. As a result, in F-measure, the top two algorithms, PPSampler2 and NWE, are comparable.

**Table 4:** The matching results on size three.

	MCL	MCODE	DPClus	CMC	COACH	RRW	NWE	PPSampler1	PPSampler2
#protein	456	162	120	616	60	309	162	180	247.80 ± 13.56
#cluster	152	54	40	216	20	103	54	60	82.60 ± 4.52
*N_pc_*	13	6	3	5	0	45	43	32	47.60 ± 4.18
*N_kc_*	55	4	38	16	52	48	50	48	69.80 ± 0.60

**Figure 6:**
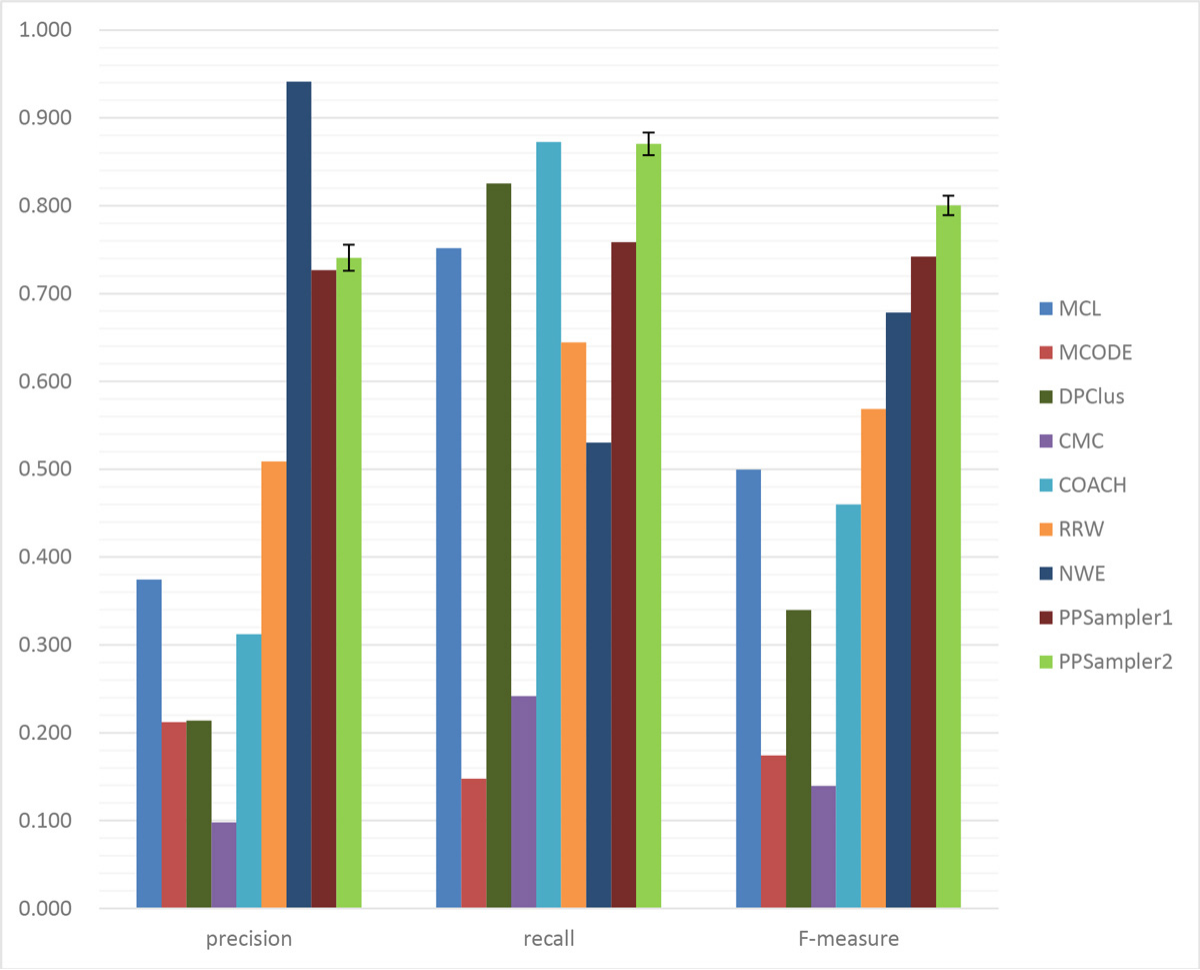
**Performance comparison on protein complexes of size four or more**.

The matching results on size four or more is shown in Table 5 and the performance is given in Figure 6. In precision, the best algorithm is NWE, followed by PPSampler2. In recall, PPSampler2 and COACH are comparable. In F-measure, PPSampler2 is the best, followed by PPSampler1. Thus, it turns out that even for these large sizes, PPSampler2 is shown to be empirically superior to the other algorithms.

**Table 5:** The matching results on size four or more.

	MCL	MCODE	DPClus	CMC	COACH	RRW	NWE	PPSampler1	PPSampler2
#protein	4951	2264	4799	5795	4052	283	200	1563	1542.90 ± 15.27
#cluster	497	99	884	756	1333	57	34	161	209.90 ± 2.81
Avg. size	9.96	22.87	7.09	25.84	13.45	5.00	5.88	9.71	7.35 ± 0.10
*N_pc_*	186	21	189	74	416	29	32	117	155.50 ± 4.36
*N_kc_*	112	22	123	36	130	96	79	113	129.70 ± 1.90

### Evaluation by Gene Ontology

It is reasonable to suppose that the list of protein complexes recorded in databases are still incomplete. This assumption indicates potential protein complexes. Under the assumption, statistically significant clusters by GO which are unmatched with any known complexes are good candidates for potential protein complexes.

The number of statistically significant clusters unmatched with any known complexes is shown in Table 6. The row of #unmat. shows the number of predicted clusters unmatched with any known complexes. Each of the following rows gives the number of statistically significant ones among the unmatched clusters on the corresponding GO aspect, biological process (BP), cellular component (CC), or molecular function (MF). It can be found that the proportions of such clusters given by PPSampler2 are 82%, 51%, and 55% on the biological process, cellular component, and molecular function aspects of GO, respectively. The most related aspect to protein complexes would be biological process as they are considered to work in particular biological processes in the form of protein complexes. In fact, 82% of the predicted clusters unmatched with any known complexes are determined to be statistically significant on the biological process aspect of Gene Ontology. Those predicted clusters are good candidates for potential protein complexes.

**Table 6:** Statistically significant clusters unmatched with any known complexes.

	MCL	MCODE	DPClus	CMC	COACH	RRW	NWE	PPSampler1	PPSampler2
#unmat.	674	129	733	899	937	1788	516	162	158
BP	203	41	220	356	611	322	215	107	130
CC	137	33	155	267	530	214	142	70	80
MF	150	34	163	276	510	191	127	75	87

The row of #unmat. shows the number of predicted clusters unmatched with any known complexes. Each of the following rows gives the number of statistically significant ones among the unmatched clusters on the corresponding GO aspect, biological process (BP), cellular component (CC), or molecular function (MF).

**Figure 7:**
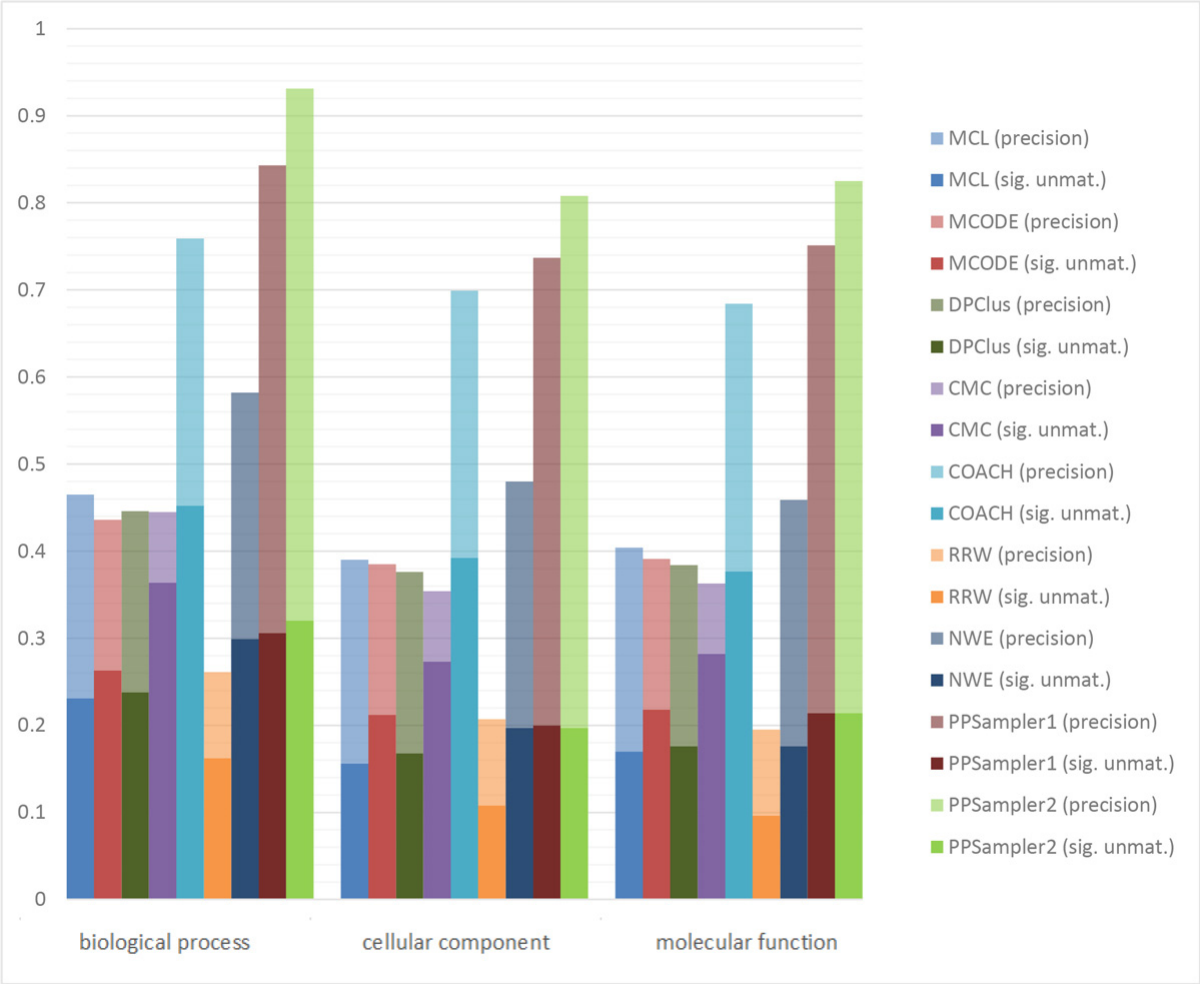
**Extended precision by GO**. The total height of a bar is equal to the fraction of the sum of the number of clusters matched with at least one known complex and the number of statistically significant clusters unmatched with any known complexes to the number of predicted clusters. The lower part of a bar is proportional to the number of statistically significant clusters unmatched with any known complexes. The upper part is equivalent to the precision.

Figure 7 shows extended precision by an aspect of GO. The total height of a bar is equal to the fraction of the sum of the number of clusters matched with at least one known complex and the number of statistically significant clusters unmatched with any known complexes to the number of all predicted clusters. This measure is called the extended precision by an aspect of GO. The lower part of a bar is proportional to the number of statistically significant clusters unmatched with any known complexes. The upper part is equivalent to the precision. It can be seen that PPSampler2 outperforms the others in every aspect of GO. Especially, in the biological process aspect, the extended precision of PPSampler2 is 92%. This implies that most of the predicted clusters by PPSampler2 are biologically meaningful.

### Random clusters

The evaluation of randomly generated clusters of proteins is also carried out to see how meaningful the evaluation of clusters predicted from the original PPIs is. A random partition of all proteins is generated by shuffing the input PPIs in the following way. The text file of WI-PHI has three columns, corresponding to an interactor, the other interactor, and their weight. Each column is permutated randomly. PPSampler2 is applied to the resulting random PPIs with the default parameter set. This process is repeated three times and their performance scores are almost the same. Thus, one of them is picked up and is summarized as follows.

The number of proteins within predicted clusters of size two or more is 2012. The number of those predicted clusters is 731, and among them only one cluster is matched with a known complex. Thus, the precision score is 0.001. The number of complexes matched with some predicted clusters is also one. Then the recall score is 0.002, and the resulting F-measure score is 0.002. The numbers of statistically significant clusters on the GO aspects, biological process, cellular component, and molecular function are 53, 25, and 34, respectively, and their fractions to the number of predicted clusters are 7.3, 3.4, and 4.7%, respectively. Thus, these results imply that the predicted clusters from the original PPIs of WI-PHI are very meaningful.

### Robustness

In this section, we assess the robustness of PPSampler2 to some important parameters of it.

**Figure 8:**
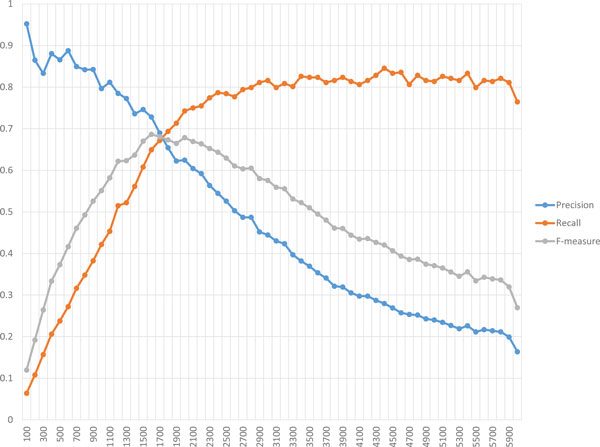
**Robustness of PPSampler2 to *λ***. The scores of precision, recall, and F-measure are plotted on the horizontal axis representing values of *λ*.

The first parameter is *λ*, representing a target number of proteins within clusters of size two or more, used in the scoring function, *g*_3_. The values of *λ *applied here are ranged from 100 to 6000 with 100 increments in between. For each of them, a single execution of PPSampler2 is carried out. The resulting performance is shown in Figure 8. The precision line almost monotonically decreases according to the decrease of *λ*. On the other hand, the recall line increases up to about 0.8 when the value of *λ *moves up to 3000 from 100, and is then saturated at the same level thereafter. This observation on recall would imply the incompleteness of known complexes of CYC2008. If more true complexes were available, the recall line would continue to increase more and the precision line would decline more gradually. Recall that an F-measure score is calculated from those of precision and recall. As can be seen, the F-measure line has a unique peak and the F-measure scores at *λ *= 1100*, . . . *, 2800 are more than 0.6. Thus, the following implication can be obtained. When candidates for yet-to-be-discovered protein complexes are needed to be obtained, the value of *λ *should be set to be larger to some extent.

**Figure 9:**
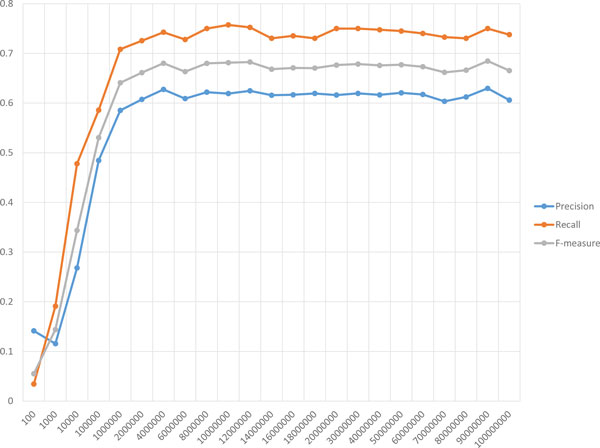
**Robustness of PPSampler2 to *L***. The scores of precision, recall, and F-measure are plotted on the horizontal axis representing values of *L*.

The second parameter to be considered here is *L*, the number of iterations of PPSampler2. Recall that the default value of *L *is set to be 2 × 10^6^. The following values are used as *L*: 10*i *for ^*i *^= 2*, . . . *, 6, *j *× 10^6 ^for *j *= 2, 4*, . . . *, 20, and *k *× 10^7 ^for *k *= 3, 4*, . . . *, 10. The result is shown in Figure 9. As can be seen, every line is saturated from *L *= 2 × 10^6^. Thus, the default value of *L *is set to be that value.

### PPSampler2 without regulation of frequency of sizes of predicted clusters

**Figure 10:**
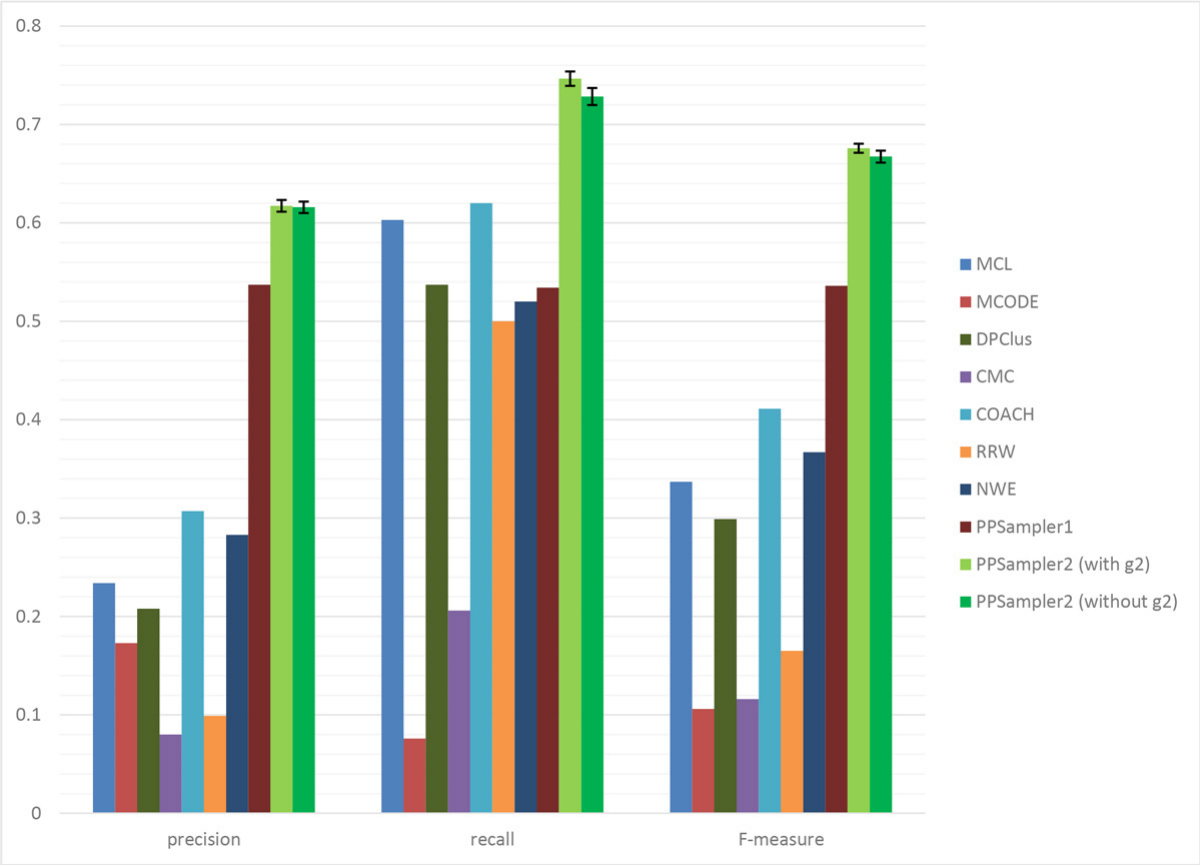
**Performance of PPSampler2 without *g*_2_**.

Recall that the scoring function, *g*_2_(*C*), regulates the frequency of sizes of predicted clusters in *C *so that it obeys a power-law distribution. As we mentioned in the Background section, even without *g*_2_, PPSampler2 can generate almost the same outputs as with *g*_2_. Performance comparison of PPSampler2 with and without *g*_2 _can be found in Figure 10. The latter case is also averaged over 10 outputs. The performances of the other algorithms are again shown there as a reference. It can be confirmed that in every performance measure, both are comparable although the score without *g*_2 _is slightly lower than that with *g*_2_.

**Figure 11:**
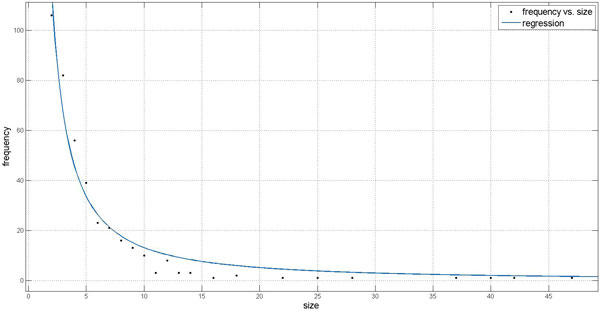
**Regression curve to the frequency of sizes of the predicted clusters**. The horizontal axis represents the cluster size. The vertical axis shows the frequency of clusters of a particular size.

It is interesting to see how much the frequency of sizes of clusters predicted by PPSampler2 without *g*_2 _obeys a power-law distribution. Among the ten outputs of PPSampler2 without *g*_2_, the most typical one, whose F-measure score is the closest to the mean of the ten F-measure scores, is picked up. The regression curve to the frequency of sizes of the predicted clusters of the output is shown in Figure 11. The expression of regression curve is 295.9 × *i*^−1.352 ^with a small root-mean-square error (RMSE) 5.999 where *i *is a cluster size. Thus, we can say that even without *g*_2_, PPSampler2 can generate clusters whose size distribution obeys a power-law distribution. Probably, this outcome is realized due to the scoring function of a cluster *c*,

$$g_{\text{1}}\left(c\right)=\underset{u,v\left(\neq u\right)\in \;c}{\sum }\frac{w\left(u,\;v\right)}{\sqrt{\left|c\right|}}.$$

Suppose that instead of $\sqrt{\left|c\right|}$, the denominator was $\frac{|c|\left(|c|-1\right)}{2}$, which is the total number of possible interactions within *c*. Under this assumption, *g*_1_(*c*) is equivalent to the mean of the weights of all pairs of proteins within *c*. This fact implies that clusters of size two with an interaction whose weight is higher than those of the neighboring interactions are likely to be formed because for such a size-two cluster, if a neighboring protein is added to the cluster, the averaged weight is lower than that of the size-two cluster. Thus, if the denominator is $\frac{|c|\left(|c|-1\right)}{2}$, *g*_1 _cannot make the frequency of sizes of predicted clusters obey a power-law distribution. On the other hand, if the denominator of *g*_1 _is $\sqrt{\left|c\right|}$, clusters are allowed to be larger to some extent. Namely, even if the weights of neighboring interactions are lower than that of an interaction of a size-two cluster, *g*_1 _can become larger by adding another proteins. This would be the mechanism of finding a set of clusters whose size distribution is a power-law distribution.

## Conclusions

We have proposed a new protein complex prediction method, PPSampler2, by improving the scoring functions and proposal distribution of PPSampler1. The performance of PPSampler2 is superior to other methods. Especially, 92% of the predicted clusters are either matched with known complexes or statistically significant on the biological process aspect of GO. Namely, most of the predicted clusters by PPSampler2 are biologically reliable. Thus, PPSampler2 is useful to find good candidates for potential protein complexes.

## Competing interests

There is no other financial or non-financial competing interests.

## Authors' contributions

CKW analyzed outputs of PPSampler2 and drafted part of the Result section. OM designed the computational methods, implemented the computer programs, performed most of the computational experiments, analyzed the results, and drafted the paper. Both authors read and approved the final manuscript.

## Supplementary Material

Additional file 1 **Example of known protein complexes perfectly detected by PPSampler2**. All of the known protein complexes perfectly detected by PPSampler2 and not by the other tools are extracted. For each of those known protein complexes, the best overlap ratio obtained by each tool is given. Click here for file

## Declarations

The publication fee was funded by the Institute of Mathematics for Industry at Kyushu University.

This article has been published as part of *BMC Systems Biology *Volume 7 Supplement 6, 2013: Selected articles from the 24th International Conference on Genome Informatics (GIW2013). The full contents of the supplement are available online at http://www.biomedcentral.com/bmcsystbiol/supplements/7/S6.
