# Supplementary material for: PPSampler2: Predicting protein complexes more accurately and efficiently by sampling
Source: BMC Syst Biol. 2013 Dec 13;7(Suppl 6):S14. doi: 10.1186/1752-0509-7-S6-S14 (PMC4029527; doi:10.1186/1752-0509-7-S6-S14)
Supplement: Additional file 1 — Example of known protein complexes perfectly detected by PPSampler2. All of the known protein complexes perfectly detected by PPSampler2 and not by the other tools are extracted. For each of those known protein complexes, the best overlap ratio obtained by each tool is given. [file 1752-0509-7-S6-S14-S1.PDF]

| Size | Complex name | MCL | MCODE | DPClus | CMC | COACH | RRW | NWE | PPSampler1 | PPSampler2 |
|---|---|---|---|---|---|---|---|---|---|---|
| 2 | 6-phosphofructokinase complex | 0 | 0 | 0 | 0 | 0.45 | 0 | 0.82 | 0 | 1 |
| | Ribosome-associated complex (RAC) | 0.50 | 0 | 0 | 0.47 | 0 | 0 | 0.82 | 0 | 1 |
| | Fig4p/Vac14p complex | 0.54 | 0 | 0 | 0.53 | 0.71 | 0 | 0.82 | 0 | 1 |
| | anthranilate synthase complex | 0.71 | 0 | 0 | 0 | 0 | 0 | 0.82 | 0 | 1 |
| 3 | Rad17p/Ddc1p/Mec3p complex | 0.71 | 0 | 0 | 0 | 0.52 | 0.82 | 0.67 | 0.77 | 1 |
| | chromatin silencing complex | 0.61 | 0 | 0 | 0 | 0.61 | 0 | 0 | 0.65 | 1 |
| | karyopherin docking subcomplex of the Nuclear Pore Complex (NPC) | 0.61 | 0 | 0.65 | 0.61 | 0 | 0 | 0 | 0 | 1 |
| | DNA polymerase delta complex | 0.87 | 0 | 0.87 | 0 | 0.47 | 0.82 | 0.87 | 0.82 | 1 |
| | NatA complex | 0.65 | 0 | 0 | 0 | 0.61 | 0.82 | 0.87 | 0 | 1 |
| | Kel1p/Kel2p/Lte1p complex | 0.77 | 0 | 0 | 0 | 0 | 0.82 | 0 | 0.71 | 1 |
| | Apg12p/Apg5p/Apg16 multimeric complex | 0.50 | 0 | 0.52 | 0 | 0.55 | 0.82 | 0.87 | 0.87 | 1 |
| | Polzeta-Rev1p complex | 0.71 | 0 | 0 | 0 | 0 | 0.82 | 0.67 | 0 | 1 |
| | methionyl glutamyl tRNA synthetase complex | 0.71 | 0 | 0 | 0 | 0 | 0 | 0.87 | 0.50 | 1 |
| | RNA Degradation Complex | 0.61 | 0 | 0 | 0.58 | 0 | 0.82 | 0.67 | 0 | 1 |
| | Atg20p/Snx4p/Snx41p complex | 0.65 | 0 | 0.71 | 0 | 0 | 0.82 | 0.67 | 0 | 1 |
| | RNA polymerase I transcription factor complex | 0.55 | 0.61 | 0.77 | 0 | 0.87 | 0.82 | 0.87 | 0 | 1 |
| 4 | alpha DNA polymerase:primase complex | 0.75 | 0 | 0.52 | 0 | 0.55 | 0.71 | 0.75 | 0.75 | 1 |
| | alpha,alpha-trehalose-phosphate synthase complex (UDP-forming) | 0.50 | 0 | 0.50 | 0 | 0.53 | 0 | 0.89 | 0.58 | 1 |
| | ARGR complex | 0.55 | 0 | 0.82 | 0 | 0.63 | 0.71 | 0.75 | 0.87 | 1 |
| | Sec62p/Sec63p complex | 0.76 | 0 | 0.82 | 0 | 0.82 | 0.87 | 0.75 | 0.82 | 1 |
| | Ndc80p complex | 0.67 | 0 | 0.76 | 0 | 0.76 | 0.87 | 0 | 0.82 | 1 |
| | Nsp1p complex | 0.55 | 0 | 0.55 | 0 | 0.63 | 0.87 | 0 | 0 | 1 |
| | AP-3 adaptor complex | 0.71 | 0.87 | 0.71 | 0 | 0.82 | 0.71 | 0.58 | 0.71 | 1 |
| 5 | eEF1 | 0.67 | 0 | 0.68 | 0 | 0.73 | 0.63 | 0.52 | 0.52 | 1 |
| | AP-1 adaptor complex | 0.51 | 0 | 0.71 | 0 | 0.71 | 0.77 | 0 | 0 | 1 |
| | alpha-1,6-mannosyltransferase complex (Anp1p/Mnn9p) | 0.53 | 0 | 0.71 | 0 | 0.75 | 0.77 | 0 | 0.62 | 1 |
| 6 | Nup84p complex | 0.52 | 0 | 0.71 | 0 | 0.72 | 0.91 | 0 | 0.46 | 1 |
| | Signal recognition particle (SRP) | 0.82 | 0.77 | 0.82 | 0 | 0.77 | 0.82 | 0.93 | 0.93 | 1 |
| | prefoldin complex | 0.65 | 0 | 0.87 | 0 | 0.77 | 0 | 0.47 | 0.73 | 1 |
| 7 | Arp2/3 protein complex | 0.61 | 0 | 0.68 | 0 | 0.61 | 0.65 | 0 | 0.61 | 1 |
| | Set3p complex | 0.71 | 0 | 0.73 | 0 | 0.84 | 0.53 | 0.86 | 0.94 | 1 |
| | nucleotide-excision repair factor 3 complex | 0.54 | 0 | 0.84 | 0 | 0.71 | 0.53 | 0.57 | 0.57 | 1 |
| 9 | oligosaccharyl transferase complex | 0.77 | 0.52 | 0.80 | 0 | 0.75 | 0.88 | 0 | 0.82 | 1 |
| 14 | 20S proteasome | 0.78 | 0 | 0.84 | 0 | 0.91 | 0.53 | 0 | 0.69 | 1 |
| 25 | Kornberg's mediator (SRB) complex | 0.88 | 0.50 | 0.93 | 0 | 0.93 | 0.53 | 0 | 0.83 | 1 |
